# The consequences of the COVID-19 pandemic on the health and wellbeing of the youth: a systematic review: Mark Bosmans

**DOI:** 10.1093/eurpub/ckac129.480

**Published:** 2022-10-25

**Authors:** M Bosmans, E Marra, C Baliatsas, M de Vetten-Mc Mahon, M Dückers

**Affiliations:** 1 Disasters and Environmental Hazards, NIVEL, Utrecht, Netherlands; 2 Centre for Environmental Safety and Security, RIVM, Bilthoven, Netherlands; 3 Impact, ARQ National Psychotrauma Centre, Diemen, Netherlands; 4 Faculty of Behavioural and Social Sciences, University of Groningen, Groningen, Netherlands

## Abstract

**Introduction:**

The coronavirus outbreak and resulting restrictive measures have a major impact on population health. Research results suggest that the impact on the youth is especially great. In this review of international literature we investigated the impact of the COVID pandemic on the health and wellbeing of the youth, with a special emphasis on identifying vulnerable groups.

**Methods:**

This review looked at the impact on five domains; physical health, (health)care needs, mental health, social effects and other effects. We also identified protective and risk factors. We searched the databases of Pubmed, PsychInfo and Embase for longitudinal empirical studies in May 2021.

**Results:**

For all topics together, 145 papers were included. Results show that the corona crisis had a negative impact on the physical and mental health of the youth. Many young people exercised less, ate less healthy and suffered more from depression, anxiety and loneliness. Young people who already had mental or physical health problems were more vulnerable and experienced more negative results of the corona crisis. The crisis worsened existing problems. Other factors that exacerbate problems are poverty and poor family functioning. The impact is the largest for youths with multiple problems. At the same time, young people seem resilient. Many had no or few health problems during the studied period (roughly the first year of the pandemic), or their health problems decreased when restrictions were lifted.

**Discussion:**

More attention is needed for preventive interventions that prevent health problems, promote health and that support the youth to maintain and build their resilience. Special attention should be paid to vulnerable groups when targeting these interventions.

